# Supplementary material for: Gene Therapy with Enterovirus 3 C Protease: A Promising Strategy for Various Solid Tumors
Source: Nat Commun. 2025 May 8;16:4298. doi: 10.1038/s41467-025-59440-8 (PMC12062448; doi:10.1038/s41467-025-59440-8)
Supplement: Supplementary file 1 — Supplementary Information [file 41467_2025_59440_MOESM1_ESM.pdf]

Supplementary Information of Gene Therapy with Enterovirus 3C Protease: A Promising Strategy for Various Solid Tumors

Authors: Xiaotong Yang[1,2,#], Wei Li[1,#], Shaokang Yang[1,#], Zhuang Wang[1], Jiye Yin[3], Wenhao Zhang[3], Huimin Tao[1,2], Siqi Li[1], Xiaojia Guo[1], Qingsong Dai[1], Weiyan Zhu[1], Yuexiang Li[1], Xintong Yan[1,4], Chongda Luo[1,4], Jiazheng Li[1,4], Sichen Ren[1,4], Ping Wang[1,4], Yunfeng Shao[1,4], Yan Luo[1,4], Zhenyang Li[1], Jingjing Yang[1,4], Zhijie Chang[2], Ruiyuan Cao[1,*], Song Li[1,4*], Wu Zhong[1,*]

Affiliations:

[1] National Engineering Research Center for the Emergency Drug, Beijing Institute of Pharmacology and Toxicology, Beijing 100850, China

[2] School of Basic Medical Sciences, Tsinghua University, Beijing 100084, China

[3] National Beijing Center for Drug Safety Evaluation and Research, Beijing Institute of Pharmacology and Toxicology, Beijing 100850, China

[4] Song Li's Academician Workstation of Hainan University (School of Pharmaceutical Sciences), Yazhou Bay, Sanya, 572000, China.

[#] These authors contributed equally to this work

[*]Corresponding Authors:
Wu Zhong, E-mail: zhongwu@bmi.ac.cn;
Song Li, E-mail: lis.lisong@gmail.com;
Ruiyuan Cao, E-mail: 21cc@163.com.

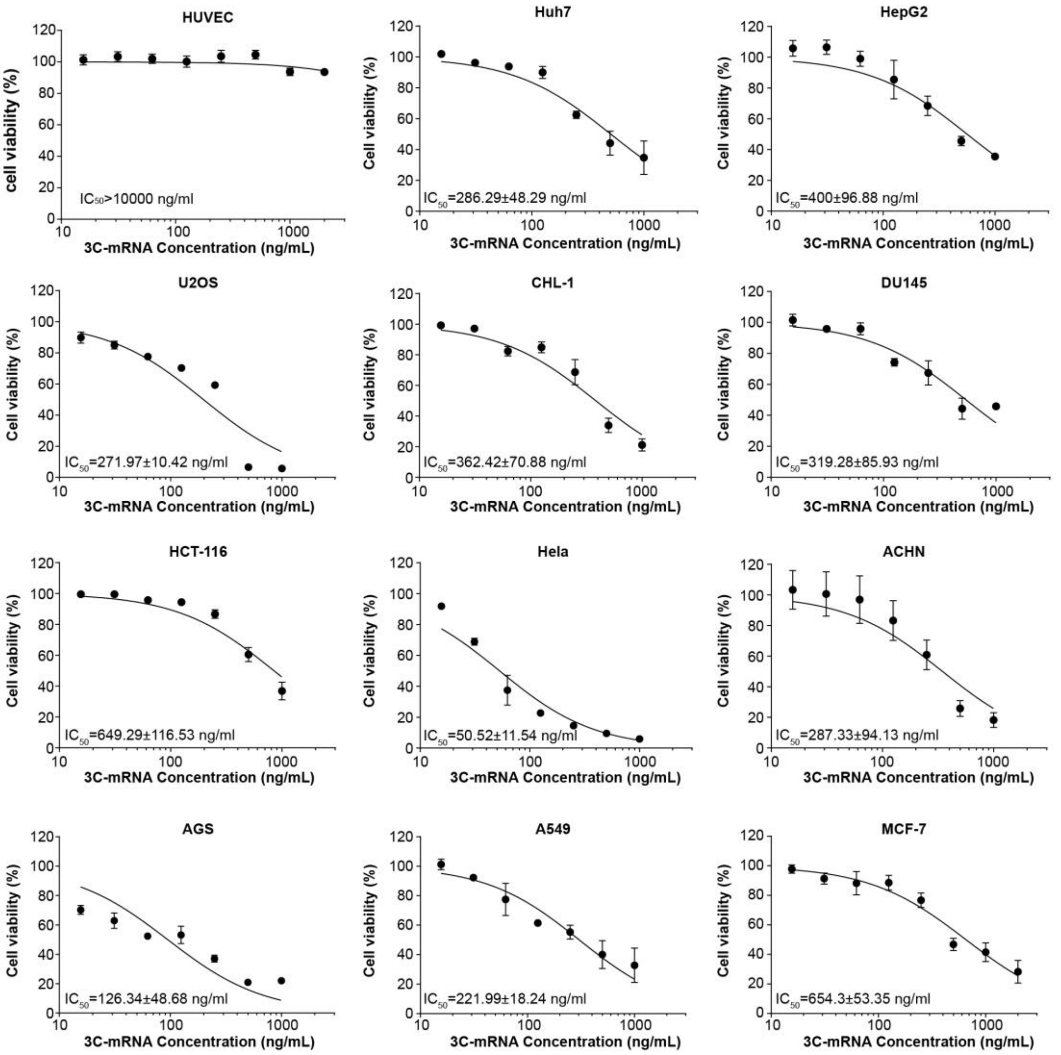

Supplementary Fig. 1| 3C-mRNA has broad-spectrum antitumor activity *in vitro*. Cell viability was assessed in the following cell lines after transfection with 3C-mRNA at 998.4 ng/mL, 499.2 ng/mL, 249.6 ng/mL, 124.8 ng/mL, 62.4 ng/mL, 31.2 ng/mL and 15.6 ng/mL: Huh7 and HepG2 hepatocellular carcinoma (HCC), U2OS osteosarcoma, CHL-1 melanoma, DU145 prostate cancer, HCT-116 human colorectal carcinoma, Hela cervical cancer, ACHN human renal adenocarcinoma, AGS gastric cancer and A549 lung cancer cells and Human umbilical vein endothelial cells (HUVECs) were used as a non-tumor control. The data are presented as the means ± SDs. n=3 independent biological samples. Source data are provided as a Source Data file.

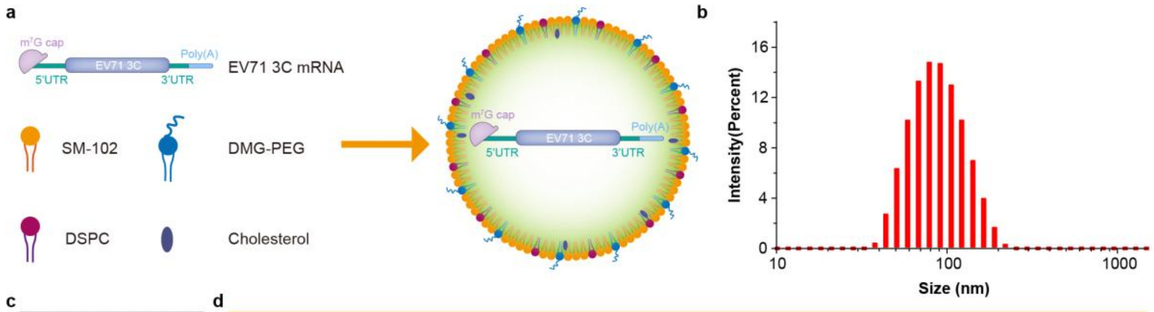

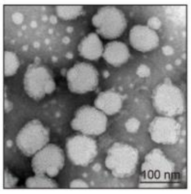

d

Size (nm)	PDI	ζPotential (mV)	EE
82.76	0.149	-3.745	94.41%

Supplementary Fig. 2| Encapsulation and characterization of 3C-LNPs. (a) Schematic illustration of the 3C-mRNA preparation. The molar ratio of the LNPs was SM-102: Distearoylphosphatidylcholine (DSPC): Cholesterol: Distearoylphosphatidyl ethanolamine-polyethylene glycol (DMG-PEG)=50:10:38.5:1.5. LNPs were produced by mixing an ethanol–lipid mixture with an mRNA in a sodium citrate acidizing buffer using a microfluidic mixer. (b) Representative intensity-size graph of 3C-LNPs measured by the dynamic light-scattering method. (c) Representative Cryo-TEM image of 3C-LNPs) from 3 independent experiments; scale bar, 100 nm. (d) The size, polymer dispersion index (PDI), zeta potential and encapsulation efficiency of 3C-LNPs were evaluated. The data are presented as the means ± SDs of five independent preparations. Source data are provided as a Source Data file.

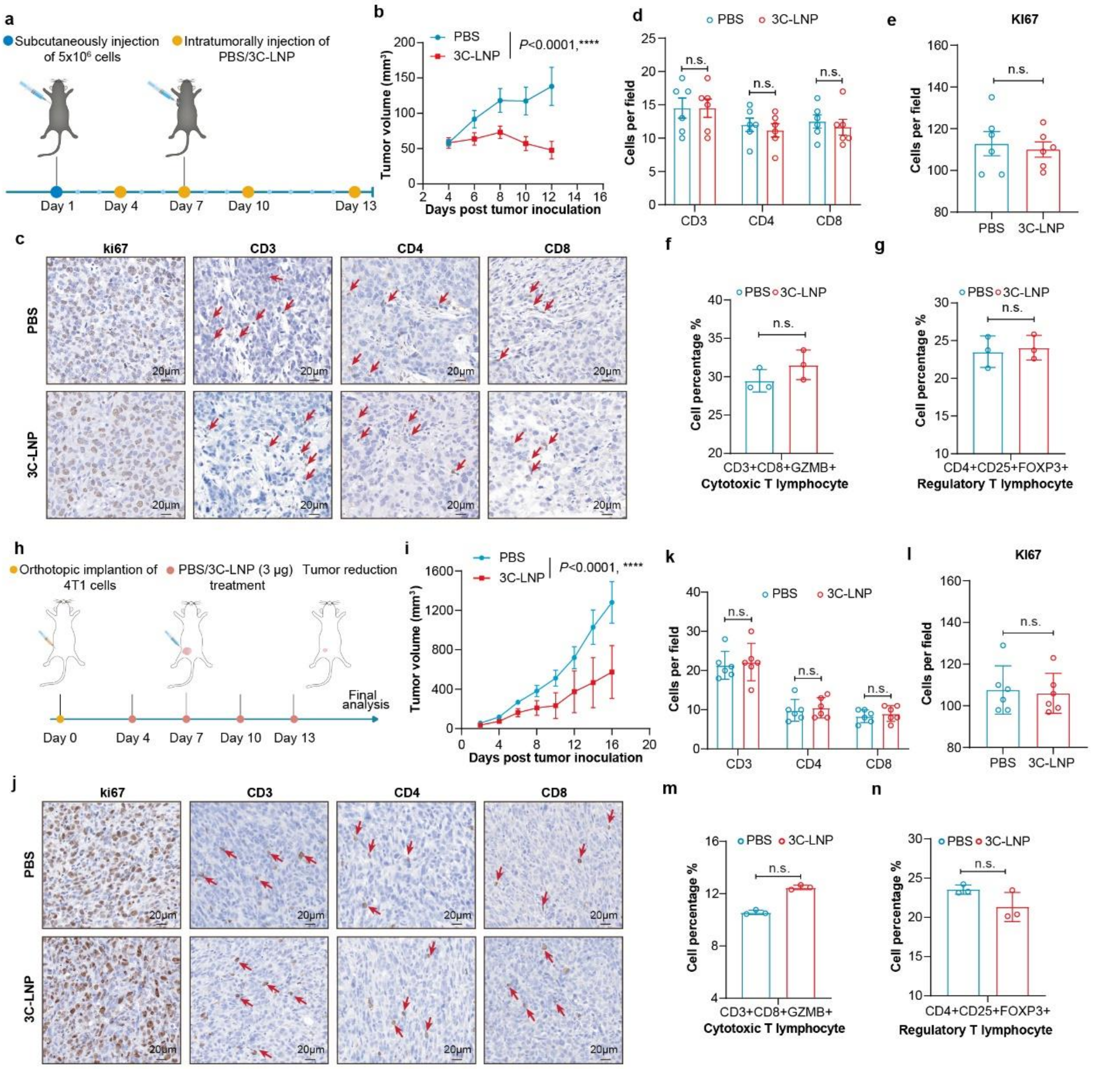

Supplementary Fig. 3| The antitumor activity of 3C-LNPs is independent of T cell-mediated antitumor immune response. (a) Timeline of tumor implantation and treatment schedule in the subcutaneous tumor model of C57BL/6 mice. Tumor-bearing mice were subcutaneously injected with PBS or 3C-LNPs (3 μg) every 3 days for a total of four doses. (n=5 mice/group). (b) Tumor growth curves of the tumor-bearing mice after treatment with PBS or 3C-LNPs (n =5 mice/group). The data are presented as the means ± SEMs. Statistical differences were assessed using Two-way ANOVA with the Bonferroni multiple comparisons test. ****P < 0.0001. (c-e) and (j-l) Representative IHC staining images of tumor tissues from 3 mice/group, with quantification of CD3, CD4, and CD8+ T cells, as well as Ki-67, performed in six fields of view. The data are presented as the means ± SDs. For (d) and (k) Statistical differences were assessed using multiple t tests. For (e) and (l) Statistical differences were assessed using an unpaired t test. n.s.= not significant. (f) and (m) Quantification of cytotoxic T lymphocytes. (g) and (n) Quantifications of regulatory T lymphocytes. The data are presented as the means ± SDs. Statistical differences were assessed using an unpaired t test. n.s.=not significant. n.s.=not significant. (h) Timeline of tumor implantation and treatment schedule in the 4T1 mammary fat pad tumor model of Balb/c mice. Mice were subcutaneously injected around the tumor tissues with PBS, or 3C-LNPs (3 μg) every 3 days for a total of four doses (n=5 mice/group). (i) Tumor growth curves of the tumor-bearing mice after treatment with PBS or 3C-LNPs (n =5 mice/group). The data are presented as the means ± SDs. Statistical differences were assessed using Two-way ANOVA with the Bonferroni multiple comparisons test. ****P < 0.0001. Source data are provided as a Source Data file.

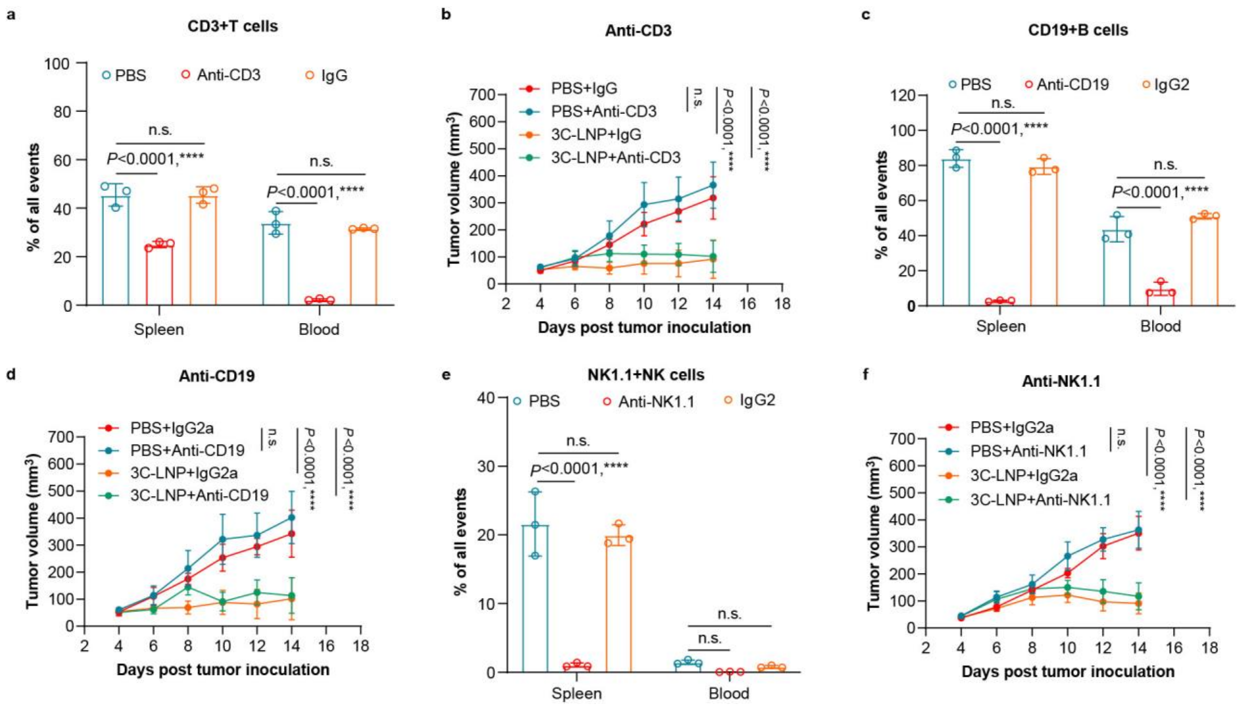

Supplementary Fig. 4| The antitumor activity of 3C-LNPs is independent of tumor immune response. Flow cytometry was employed to quantify the levels of (**a**) $CD3^+$ T cells, (c) $CD19^+$ B cells, and (e) $NK1.1^+$ NK cells in spleen and blood of mice subjected to depletion protocols, two weeks after the initiation of weekly intraperitoneal (i.p.) antibody injections. The data are presented as the means ± SDs. n =6 mice/group. Statistical differences were assessed using two-way ANOVA with the Bonferroni multiple comparisons test. n.s.=not significant. ****P < 0.0001. (b), (d) and (f) Tumor growth curves of the tumor-bearing mice after treatment with PBS combined with antibodies, PBS combined with corresponding control IgG, 3C-LNP combined with antibodies, and 3C-LNP combined with corresponding control IgG (n =6 mice/group). The data are presented as the means ± SDs. Statistical differences were assessed using two-way ANOVA with the Bonferroni multiple comparisons test. n.s.=not significant. ****P < 0.0001. Source data are provided as a Source Data file.

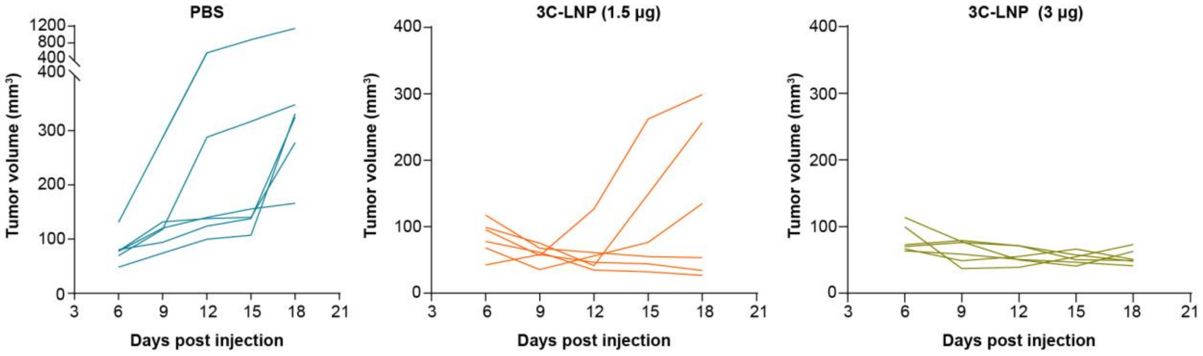

Supplementary Fig. 5| Changes in tumor volume in individual mice with orthotopic breast cancer. Growth curves of tumors from individual tumor-bearing mice after treatment with PBS, 3C-LNPs (1.5 μg), or 3C-LNPs (3 μg). n = 6 mice/group. Source data are provided as a Source Data file.

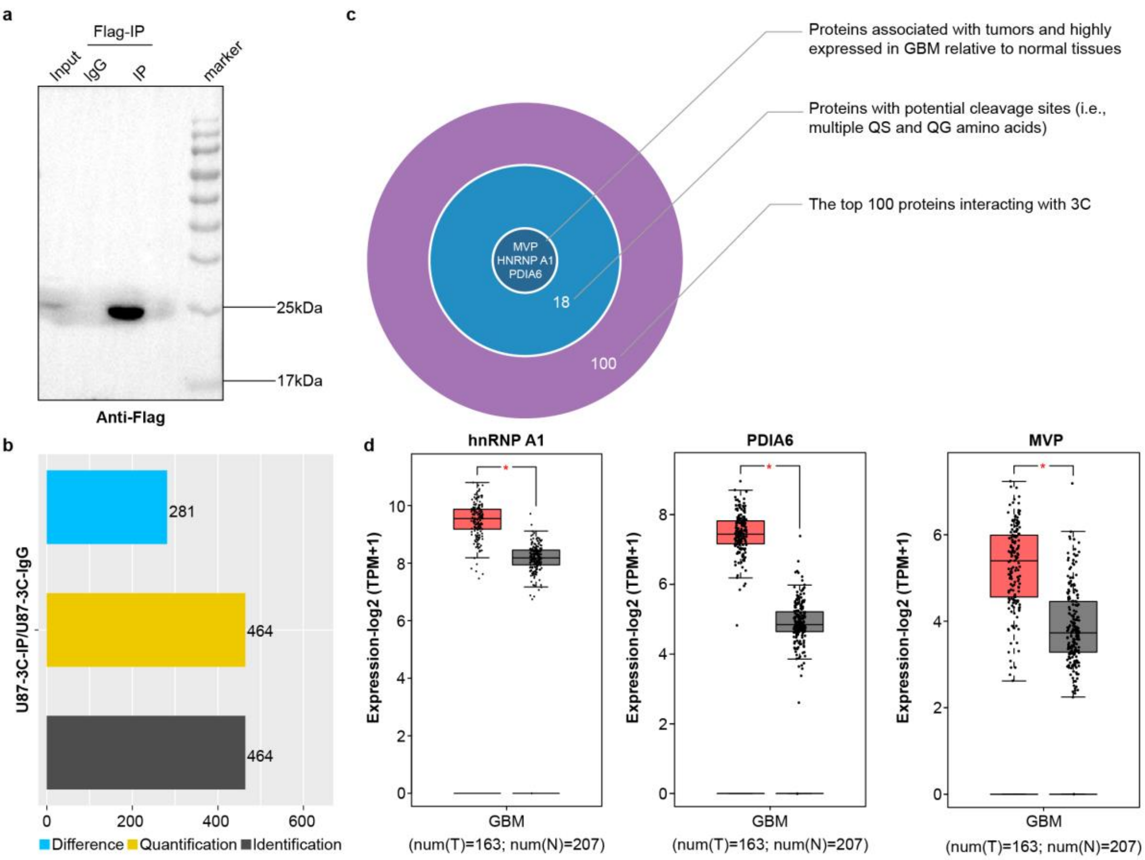

Supplementary Fig. 6| Differential abundance of hnRNP A1, PDIA6 and MVP in GBM cells. (a) U87 MG cells were transfected with a FLAG-tagged 3C expression plasmid. FLAG-IP showed that the 3C protein was sufficiently enriched for subsequent mass spectrometry identification of interacting proteins. (b) A total of 464 proteins, 281 of which were differentially abundant, were identified in the U87-3C-IP and U87-3C-IgG groups. (c) We identified 3 proteins, hnRNP A1, PDIA6, and MVP from the top 100 proteins interacting with the 3C protein and with potential $3C^{pro}$ cleavage sites. (d) Differential expression of hnRNP A1, PDIA6, and MVP in normal and GBM tumor tissues (Gene Expression Profiling Interactive Analysis database). T represents tumor tissue, N represents normal tissue. Source data are provided as a Source Data file.

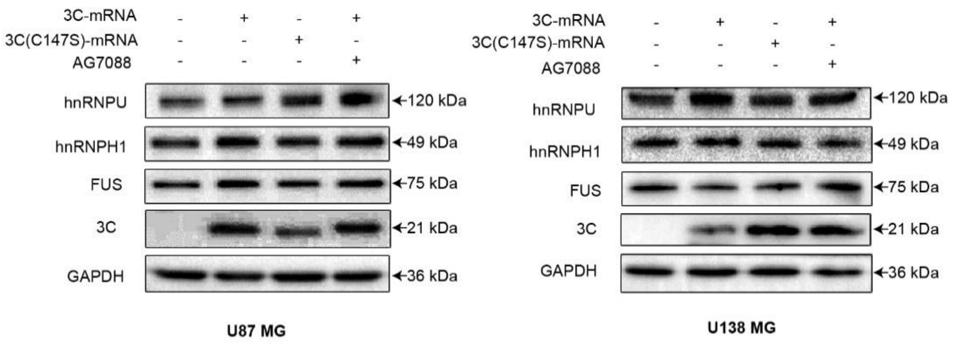

Supplementary Fig. 7| 3C protease does not affect protein expression of hnRNPU, hnRNPH1 and FUS in GBM cells. Representative western blot analysis of hnRNPU, hnRNPH1, FUS and 3C expression in U87 MG and U138 MG cells transfected with 3C-mRNA and 3C(C147S)-mRNA, and in cells transfected with 3C-mRNA and treated with AG7088 from 3 independent experiments. Source data are provided as a Source Data file.

.

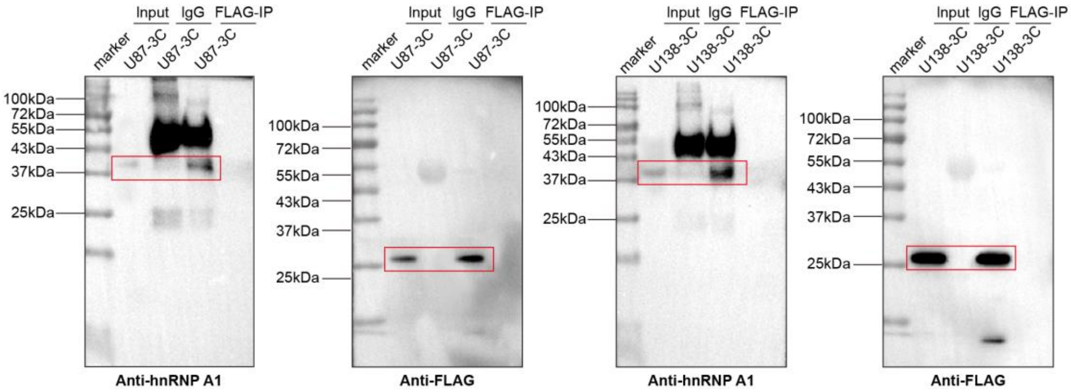

Supplementary Fig. 8| 3C protease interacts with hnRNPA1 in glioblastoma cells. Total protein from U87 MG and U138 MG cells transfected with a 3C-FLAG expression plasmid was used as the input in co-IP experiments. The FLAG-IP group comprises proteins that have coprecipitated with 3C-FLAG. Blots were incubated with anti-hnRNP A1 antibody.

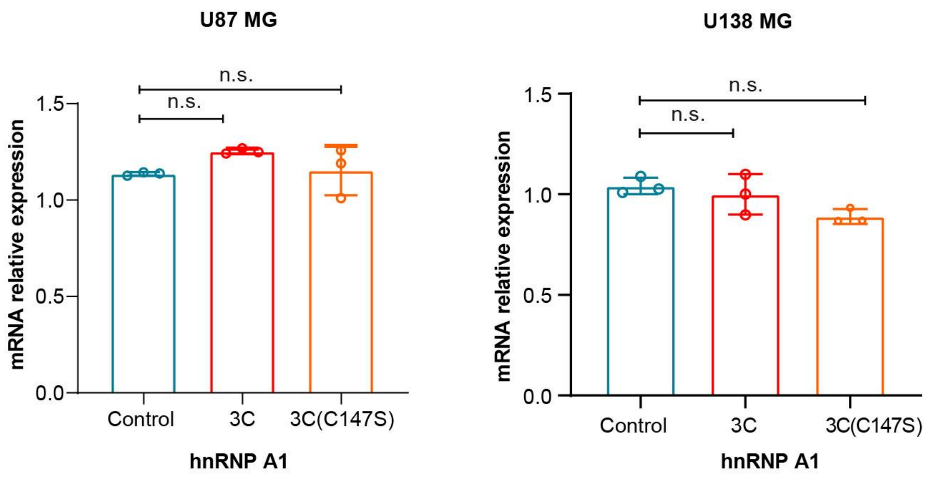

Supplementary Fig. 9| mRNA expression levels of hnRNPA1 in U87 MG and U138 MG cells. mRNA expression levels of hnRNPA1 in U87 MG and U138 MG cells following transfection with control (lipo2000), 3C-mRNA (3.5 μg), and 3C(C147S)-mRNA (3.5 μg). The mRNA relative expression was normalized to GAPDH and is presented as the mean ± SD. n = 3 cell samples/group. Statistical differences were assessed using one-way ANOVA. n.s.=not significant. Source data are provided as a Source Data file.

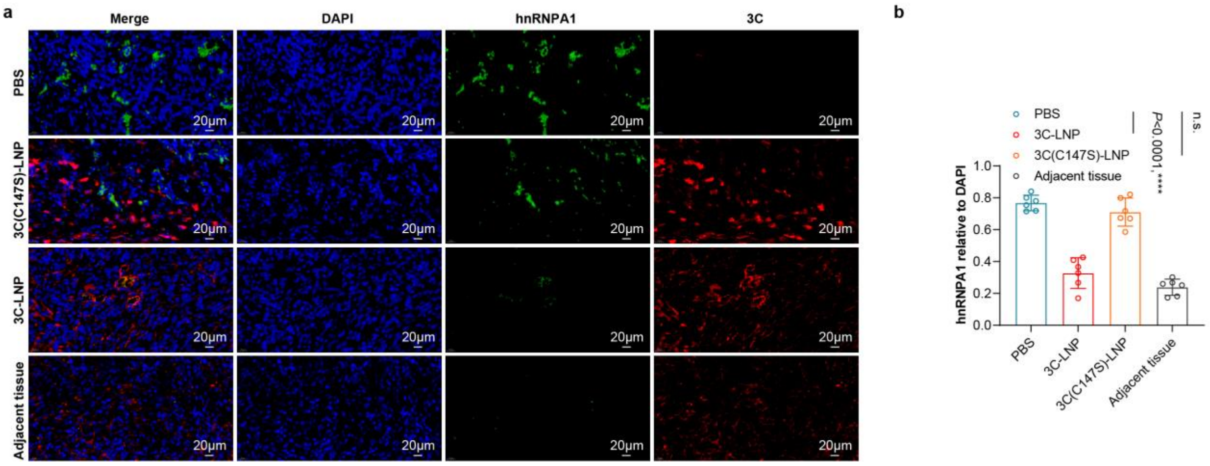

Supplementary Fig. 10| *In vivo* validation of 3C protein expression effects on hnRNPA1 levels and apoptosis induction in GBM models. (**a**) Immunofluorescence staining of tumor and adjacent sections showing the expression of hnRNPA1 (green), 3C protein (red), and cellular nuclei (DAPI, blue). The merge images illustrate co-localization. Scale bars= 20 μm. (**b**) Quantification of hnRNPA1 expression relative to DAPI. n= 6 independent fields of view. For **a**: a representative image from one of six independent fields of view in a single experiment. The data in **b** is presented as the means ± SD. Statistical differences were assessed using one-way ANOVA with the Bonferroni multiple comparisons test. n.s.=not significant. ****$P < 0.0001$. Source data are provided as a Source Data file.

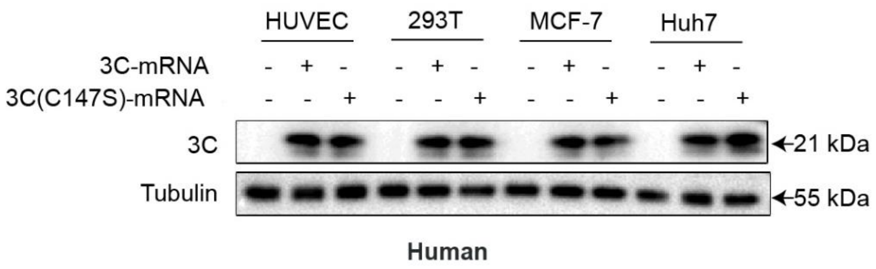

Supplementary Fig. 11| 3C expression in different human normal and cancer cells. Representative western blot analysis of 3C expression in HUVEC, 293T, MCF-7 and Huh7 cells transfected with 3C-mRNA and 3C(C147S)-mRNA from 3 independent experiments. Source data are provided as a Source Data file.

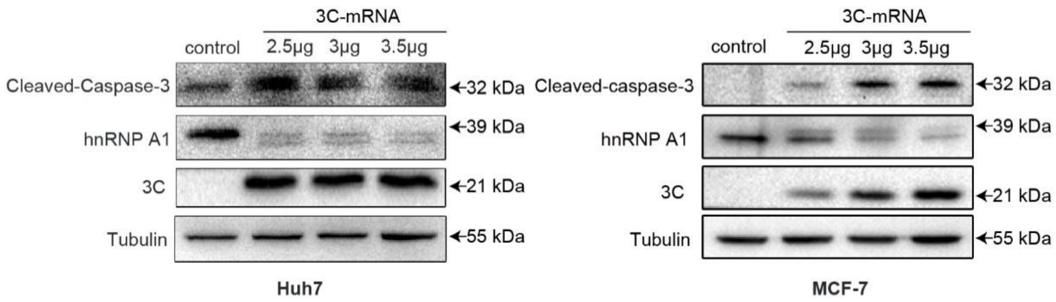

Supplementary Fig. 12| The expression levels of hnRNPA1 are significantly reduced upon 3C expression in both Huh7 and MCF7 cells. Representative western blot analysis of Cleaved-caspase-3, hnRNPA1, and 3C expression in Huh7 and MCF-7 cells transfected with different concentrations 3C-mRNA from 3 independent experiments. Source data are provided as a Source Data file.

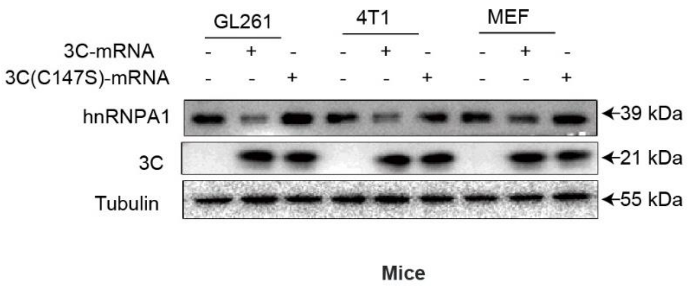

Supplementary Fig. 13| 3C expression in different mouse normal and cancer cells. Representative western blot analysis of hnRNPA1 and 3C expression in GL261, 4T1, and MEF cells transfected with 3C-mRNA and 3C(C147S)-mRNA from 3 independent experiments. Source data are provided as a Source Data file.

Supplementary Table 1. 3C-mRNA has broad-spectrum antitumor activity *in vitro*. Cell viability was measured using a CellTiter−Glo Cell Viability Assay Kit. The IC_{50} was calculated for each cell type using Origin 9.0 software. Therapeutic index (TI)=IC_{50} of HUVECs/IC_{50} of tumor cells. The data are presented as the means ± SDs. n=3 independent biological samples.

Cell line	IC_{50} (ng/mL)	Therapeutic index (TI)
**HUVEC**	> 10000	-
**U87 MG**	379.48±51.41	> 26.35
**U138 MG**	342±50.74	> 29.24
**Huh7**	286.29±48.29	> 24.99
**HepG2**	400±96.88	> 24.99
**U2OS**	271.97±10.42	> 36.77
**CHL-1**	362.42±70.88	> 27.59
**DU145**	319.28±85.93	> 31.32
**HCT-116**	649.29±116.53	> 15.40
**Hela**	50.52±11.54	> 199
**ACHN**	287.33±94.13	> 34.80
**AGS**	126.34±48.68	> 79.15
**A549**	221.99±18.24	> 45.05
**MCF-7**	654.3±53.35	> 15.29

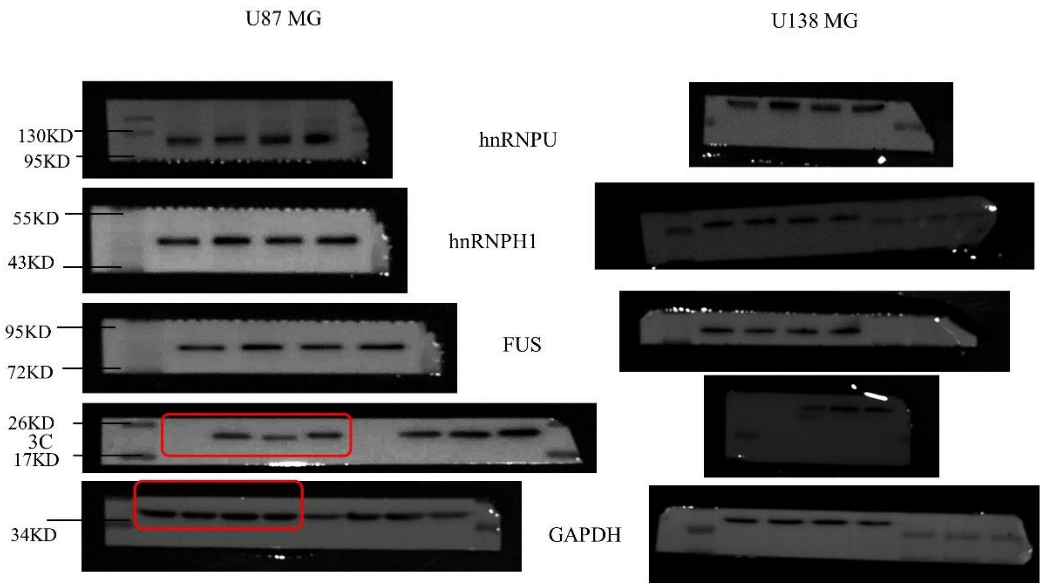

Supplementary Fig. 14| Original images for Supplementary Figure 7.

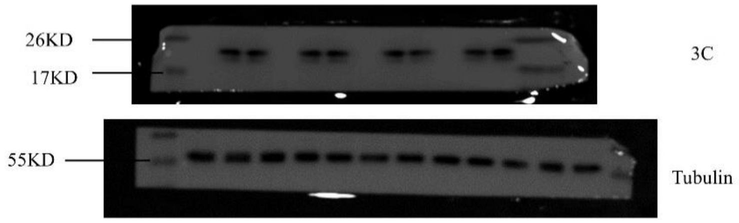

Supplementary Fig. 15| Original images for Supplementary Figure 11.

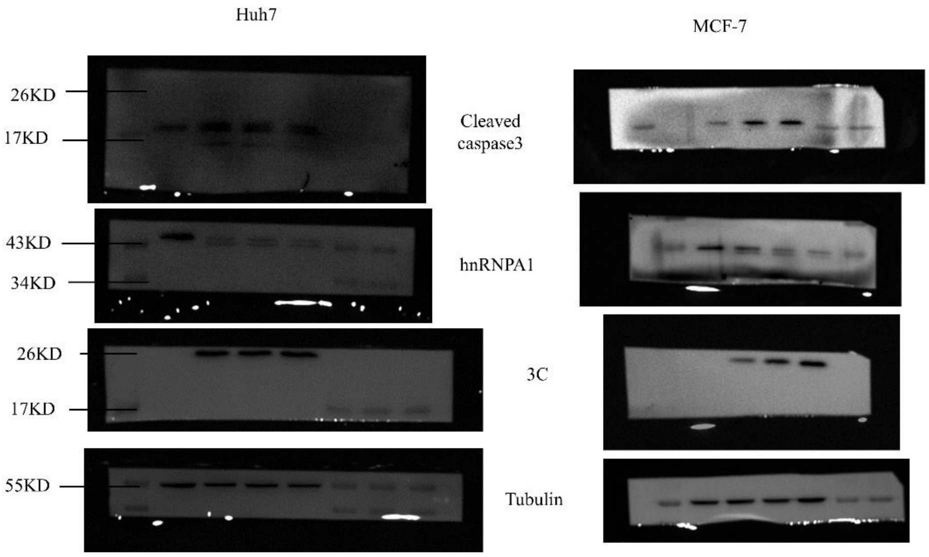

Supplementary Fig. 16| Original images for Supplementary Figure 12.

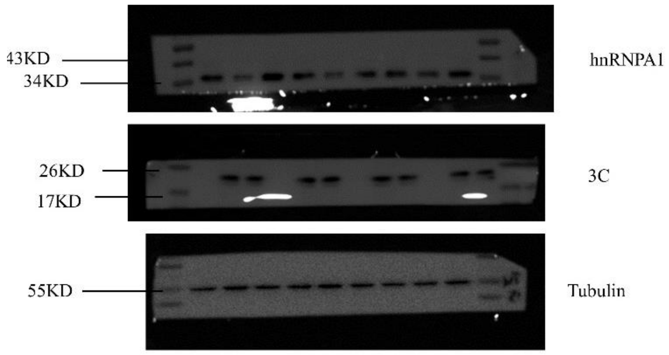

Supplementary Fig. 17| Original images for Supplementary Figure 13.
