# Supplementary material for: Gene Therapy with Enterovirus 3 C Protease: A Promising Strategy for Various Solid Tumors
Source: Nat Commun. 2025 May 8;16:4298. doi: 10.1038/s41467-025-59440-8 (PMC12062448; doi:10.1038/s41467-025-59440-8)
Supplement: Supplementary file 2 — Reporting Summary [file 41467_2025_59440_MOESM2_ESM.pdf]

Corresponding author(s): Wu Zhong, Song Li, Ruiyuan Cao

Last updated by author(s): Mar 18, 2025

Reporting Summary

Nature Portfolio wishes to improve the reproducibility of the work that we publish. This form provides structure for consistency and transparency in reporting. For further information on Nature Portfolio policies, see our Editorial Policies and the Editorial Policy Checklist.

Statistics

For all statistical analyses, confirm that the following items are present in the figure legend, table legend, main text, or Methods section.

n/a	Confirmed	
☐	☒	The exact sample size (*n*) for each experimental group/condition, given as a discrete number and unit of measurement
☐	☒	A statement on whether measurements were taken from distinct samples or whether the same sample was measured repeatedly
☐	☒	The statistical test(s) used AND whether they are one- or two-sided<br>*Only common tests should be described solely by name; describe more complex techniques in the Methods section.*
☒	☐	A description of all covariates tested
☐	☒	A description of any assumptions or corrections, such as tests of normality and adjustment for multiple comparisons
☐	☒	A full description of the statistical parameters including central tendency (e.g. means) or other basic estimates (e.g. regression coefficient) AND variation (e.g. standard deviation) or associated estimates of uncertainty (e.g. confidence intervals)
☐	☒	For null hypothesis testing, the test statistic (e.g. *F*, *t*, *r*) with confidence intervals, effect sizes, degrees of freedom and *P* value noted<br>*Give P values as exact values whenever suitable.*
☒	☐	For Bayesian analysis, information on the choice of priors and Markov chain Monte Carlo settings
☒	☐	For hierarchical and complex designs, identification of the appropriate level for tests and full reporting of outcomes
☒	☐	Estimates of effect sizes (e.g. Cohen's *d*, Pearson's *r*), indicating how they were calculated

Our web collection on statistics for biologists contains articles on many of the points above.

Software and code

Policy information about availability of computer code

Data collection	Living Image® 4.7.4 for in vivo imaging; OPTO-III for microvascular images collection; Leica Application Suit X for fluorescence imaging acquisition; EnSpire Manager for cell viability data collection; Peptides were analyzed via LC–MS/MS on a Q Exactive HF–X mass spectrometer coupled with an EASY–nLC 1200 system .
Data analysis	GraphPad Prism V_8.0.2; Flowjo 10.8.1;Microsoft Word/Excel; Adobe Illustrator 2015; CaseViewer Native Windows Application;vResolve software and ReconstructionUI; MaxQuant (V1.6.6) software

For manuscripts utilizing custom algorithms or software that are central to the research but not yet described in published literature, software must be made available to editors and reviewers. We strongly encourage code deposition in a community repository (e.g. GitHub). See the Nature Portfolio guidelines for submitting code & software for further information.

Data

Policy information about availability of data

All manuscripts must include a data availability statement. This statement should provide the following information, where applicable:

- Accession codes, unique identifiers, or web links for publicly available datasets
- A description of any restrictions on data availability
- For clinical datasets or third party data, please ensure that the statement adheres to our policy

All the data generated or analyzed during this work are included in this article and its Supplementary Information files. Source data are provided with this paper. EV 71 cleave site is available by visiting the website:https://www.ebi.ac.uk/merops/cgi-bin/pepsum?id=C03.014.

Research involving human participants, their data, or biological material

Policy information about studies with human participants or human data. See also policy information about sex, gender (identity/presentation), and sexual orientation and race, ethnicity and racism.

Reporting on sex and gender	It is not relevant as it is a preclinical study.
Reporting on race, ethnicity, or other socially relevant groupings	It is not relevant as it is a preclinical study.
Population characteristics	It is not relevant as it is a preclinical study.
Recruitment	It is not relevant as it is a preclinical study.
Ethics oversight	It is not relevant as it is a preclinical study.

Note that full information on the approval of the study protocol must also be provided in the manuscript.

Field-specific reporting

Please select the one below that is the best fit for your research. If you are not sure, read the appropriate sections before making your selection.

☒ Life sciences ☐ Behavioural & social sciences ☐ Ecological, evolutionary & environmental sciences

For a reference copy of the document with all sections, see nature.com/documents/nr-reporting-summary-flat.pdf

Life sciences study design

All studies must disclose on these points even when the disclosure is negative.

Sample size	The sample size of each experiment was provided in the corresponding figure legend. At least n=3 biologically independent repeats were performed per experimental condition in line with standards.
Data exclusions	No data were excluded.
Replication	All experiments were conducted at least triplicates independently to guarantee reproducibility.
Randomization	Samples were randomly allocated in the study.
Blinding	No blinding was used in the study due to the complicated experimental design. But the investigators were blinded to sample collection and data analysis.

Reporting for specific materials, systems and methods

We require information from authors about some types of materials, experimental systems and methods used in many studies. Here, indicate whether each material, system or method listed is relevant to your study. If you are not sure if a list item applies to your research, read the appropriate section before selecting a response.

Materials & experimental systems

n/a	Involved in the study
	☒ Antibodies
	☒ Eukaryotic cell lines
☒	Palaeontology and archaeology
	☒ Animals and other organisms
☒	Clinical data
☒	Dual use research of concern
☒	Plants

Methods

n/a	Involved in the study
☒	ChIP-seq
	☒ Flow cytometry
☒	MRI-based neuroimaging

Antibodies

Antibodies used

hnRNP A1(Cell Signaling Technology, #CST 4296), PDIA6(Proteintech, #18233-1-AP), MVP(Univ, #abs136252), APAF-1(Proteintech,#29022-1-AP), Caspase-3(Cell Signaling Technology,#CST 9662),Cleaved caspase-3(Cell Signaling Technology,#CST 9661), Cleaved caspase-3(Affnity, #AF7022), enterovirus 71 3C (genTex, #GTX630191), Tubulin(Abcam, #ab59680), Ki67(Abcam,#ab15580), FITC anti-mouse CD3ε(BioLegend,#100305), FITC anti-mouse CD4(BioLegend,#100509), PE anti-mouse CD8a Antibody(BioLegend,100707), Alexa Fluor® 647 anti-mouse CD25(BioLegend, #102019),CD3E Rabbit mAb(Abclona,#A19017), CD4 Rabbit mAb(Abcam,#ab183685),CD8A Rabbit mAb(Abclona,#A23081), Granzyme B Recombinant(BioLegend,#372203), PE anti-mouse FOXP3(BioLegend,#320007)

Validation

hnRNP A1(Cell Signaling Technology, #CST 4296): hnRNP A1 (K350) Antibody, Species reactivity: Human, Mouse, Rat, Monkey. Application: WB, IP, IF. References: PMID: 32749456. Robert, Francis et al. Nucleic acids research. 2020;48(15):8562-8575; PMID: 25313962. Yang J, Hung LH, Licht T, et al. Dev Cell. 2014;31(1):87-99;
PDIA6(Proteintech, #18233-1-AP): Species reactivity: Human, Mouse, Rat. Application: WB, IP, IHC, IF, ELISA. References: PMID: 34737357. Zhang S, Huang W, Ren L, et al. Cell Res. 2022;32(1):9-23; PMID: 30753823. Carugo A, Minelli R, Sapio L, et al. Cancer Cell. 2019;35(2):204-220.
MVP (Univ, #abs136252): Species Reactivity: Human, Mouse, Rat. Application: WB, IHC, ELISA.
APAF-1(Proteintech, #29022-1-AP): Species Reactivity: Human. Application: WB, ELISA. References: PMID: 36501024. Zhong Y, Li Z, Jin R, et al. Nutrients. 2022;14(23):4994.
Caspase-3(Cell Signaling Technology,#CST 9662): Species reactivity: Human, Mouse, Rat, Monkey. Application: WB, IP, IHC. References: PMID: 37199120. Mikdar M, Serra M, Colin E, et al. Haematologica. 2024;109(1):175-185. PMID: 38081915. Deng C, Li C, Dong X, et al. Commun Biol. 2023;6(1):1252. PMID: 37906052. Wang X, Hu Z, Zhang W, et al. J Cell Biol. 2023;222(12):e202303108.
Cleaved caspase-3(Cell Signaling Technology,#CST 9661): clone name: 5A1E, Species reactivity: Human, Mouse, Rat, Monkey. Application: WB, IP, IHC, IF. References: PMID: 37199120. Mikdar M, Serra M, Colin E, et al. Haematologica. 2024;109(1):175-185. PMID: 37038247. Zhang K, An X, Zhu Y, et al. Cancer Biol Ther. 2023;24(1):2200705.
Cleaved caspase-3((Affnity, #AF7022): Species reactivity: Human, Mouse, Rat, Bovine. Application: WB, IHC, IF. References: PMID: 35858900. Liu L, Gu M, Ma J, et al. Liu L, Gu M, Ma J, et al. PMID: 32710621. Li X, Wang J, Gong X, et al. Nucleic Acids Res. 2020;48(15):8255-8268.
enterovirus 71 3C (genTex, #GTX630191): clone name:B3, Species reactivity: Coxsackievirus A6, Enterovirus 71. Application: WB, FACS. References: PMID:28970483. Arthur Huang KY, Chen MF, Huang YC, et al. Nat Commun. 2017;8(1):762. PMID: 36106874. Fan W, McDougal MB, Schoggins JW. J Virol. 2022;96(19):e0133222.
Tubulin (Abcam, #ab59680): Species reactivity: clone name:YL1/2, Human. Application: WB. References: PMID: 34109988. Shen X, Ye Z, Wu W, et al. Int J Oncol. 2021;59(1):51. PMID: 31324765. Niemi NM, Wilson GM, Overmyer KA, et al. Nat Commun. 2019;10(1):3197.
Ki67(Abcam, #ab15580): Species reactivity: Mouse, Human. Application: IHC-P, ICC/IF. References: PMID: 36374225. Nakayama A, Roquid KA, Iring A, et al. J Exp Med. 2023;220(1): e20211628. PMID: 36961817. Wang YC, Kelso AA, Karamafrooz A, et al. Cell Rep. 2023;42(4):112296. PMID: 36539399. Cheroni C, Trattaro S, Caporale N, et al.
FITC anti-mouse CD3ε(BioLegend, #100305): Species reactivity: Mouse. References: PMID: 33930308. Kawakami R, Kitagawa Y, Chen KY, et al. Immunity. 2021;54(5):947-961.e8. PMID: 32901017. Yang W, Yu T, Huang X, et al. Nat Commun. 2020;11(1):4457.
FITC anti-mouse CD4(BioLegend,#100509): Species reactivity: Mouse. References: PMID: 30995474. Fachi JL, Felipe JS, Pral LP, et al. Cell Rep. 2019;27(3):750-761.e7. PMID: 34478639. Sun L, Kees T, Almeida AS, et al. Cancer Cell. 2021;39(10):1361-1374.e9.
PE anti-mouse CD8a Antibody(BioLegend,100707): Species reactivity: Mouse. References: PMID: 36376298. Zelenka T, Klonizakis A, Tsoukatou D, et al. Nat Commun. 2022;13(1):6954. PMID: 31031138. Paris J, Morgan M, Campos J, et al. Cell Stem Cell. 2019;25(1):137-148.e6.
Alexa Fluor® 647 anti-mouse CD25(BioLegend, #102019): Species reactivity: Mouse. References: PMID: 32553171. Siamishi I, Iwanami N, Clapes T, Trompouki E, O'Meara CP, Boehm T. Cell Rep. 2020;31(11):107756. PMID: 21768391. Abdulreda MH, Faleo G, Molano RD, et al. Proc Natl Acad Sci U S A. 2011;108(31):12863-12868.
CD3E Rabbit mAb(Abclona,#A23504): Species reactivity: Human, Mouse, Rat. References: PMID: 37316830. Wang Q, Liu P, Wen Y, et al. Mol Cancer. 2023;22(1):95. PMID: 36400933. Shi L, Li Y, Xu X, et al. Nat Metab. 2022;4(11):1573-1590.
CD4 Rabbit mAb (Abcam, #ab183685): clone name: EPR19514, Species reactivity: Mouse. References: PMID: 35794399. Zhao K, Jiang L, Si Y, Zhou S, Huang Z, Meng X. Cancer Immunol Immunother. 2023;72(1):193-209. PMID: 35931031. Vasquez EG, Nasreddin N, Valbuena GN, et al. Cell Stem Cell. 2022;29(8):1213-1228.e8.
CD8A Rabbit mAb (A23081): Species reactivity: Mouse. References: PMID: 35640059. Wang W, He J, Yang J, et al. J Med Chem. 2022;65(11):7896-7917.
Granzyme B Recombinant(BioLegend,#372203): Species reactivity: Human, Mouse. References: PMID: 35589680. Li X, Yong T, Wei Z, et al. Nat Commun. 2022;13(1):2794. PMID: 31851923. Weisberg SP, Carpenter DJ, Chait M, et al. Cell Rep. 2019;29(12):3916-3932.e5.
PE anti-mouse FOXP3(BioLegend,#320007): Species reactivity: Human, Mouse, Rat. References:PMID: 32910719. Tian J, Kou X, Wang

R, et al. Autophagy. 2021;17(9):2586-2603. PMID: 32142650. Lu Y, Zhao Q, Liao JY, et al. Cell. 2020;180(6):1081-1097.e24.

Eukaryotic cell lines

Policy information about cell lines and Sex and Gender in Research

Cell line source(s)	The following cell lines were purchased from the American Type Culture Collection (ATCC): U87 MG (HTB-14), HepG2 (HB-8065), U2OS (HTB-96), CHL-1 (CRL-9446), DU145 (HTB-81), HCT-116 (CCL-247), HeLa (CCL-2), ACHN (CRL-1611), AGS (CRL-1739), A549 (CCL-185).Huh7 cells were purchased from the National Experimental Cell Resource Sharing Platform. U138 MG, MCF-7, GL261 cells, U87 MG-luc cells (WZ01016)and Huh7-luc cells(WZ01030) were obtained from FHunan Fenghui Biotechnology Co., Ltd.HUVEC (CTCC-0804-PC) were obtained from Zhejiang Meisen Cell Technology Co., Ltd.
Authentication	All cell lines were routine validated using the Short Tandem Repeat (STR) profiling cell authentication service.The mouse-derived cell lines were subjected to species identification. For primary HUVEC cells, identification was conducted using immunofluorescence.
Mycoplasma contamination	The cells were routinely tested for mycoplasma contamination using PCR-based methods and were found to be negative.
Commonly misidentified lines (See ICLAC register)	No commonly misidentified cell lines were used.

Animals and other research organisms

Policy information about studies involving animals; ARRIVE guidelines recommended for reporting animal research, and Sex and Gender in Research

Laboratory animals	Six to eight-weeks-old female BALB/c nude mice were purchased from Sbeifer Biotechnology Co., Ltd. Seven to eight-weeks-old female C57 BL/6 J mice were purchased from Sbeifer Biotechnology Co., Ltd. Six to seven-weeks-old BALB/c mice were purchased from Sbeifer Biotechnology Co., Ltd.All the mice had unlimited access to a sterile rodent diet (pellets) and reverse osmosis-purified water and were maintained on a 12:12 h light:dark cycle at 22–24°C and 45–55% humidity.
Wild animals	No wild animals were involved in the study
Reporting on sex	The breast cancer model was constructed using female nude mice. In addition, considering that there is little correlation with sex in clinical practice, female nude mice were also used to construct glioblastoma tumor models and liver cancer models. Toxicology studies used equal numbers of male and female mice.
Field-collected samples	This study did not contain studies collected from the field.
Ethics oversight	This research complied with all relevant ethical regulations. All the experiments in this research were approved by the Institutional Animal Care and Use Committee (IACUC) of the Beijing Institute of Pharmacology and Toxicology (IACUC-DWZX-2022-622) (Approved on 14 March 2022).

Note that full information on the approval of the study protocol must also be provided in the manuscript.

Plants

Seed stocks	No plants involved.
Novel plant genotypes	No plants involved.
Authentication	No plants involved.

Flow Cytometry

Plots

Confirm that:

☒ The axis labels state the marker and fluorochrome used (e.g. CD4-FITC).

☒ The axis scales are clearly visible. Include numbers along axes only for bottom left plot of group (a 'group' is an analysis of identical markers).

☒ All plots are contour plots with outliers or pseudocolor plots.

☒ A numerical value for number of cells or percentage (with statistics) is provided.

Methodology

Sample preparation	For tumor samples, lesions of ~500 mm3 were dissected from mice and measured for total weight. Tumors were then minced using scalpels and digested with 500 U/ml Collagenase IV (Sigma), and 200 mg/ml DNAse I (Roche) per 0.3 grams of tumor weight for 30 min at 37 °C. After incubation, tumors were then passed through a 40 µm cell strainer to remove large pieces of undigested tissue.
Instrument	Novocyte flow cytometer(novocyte flow cytometer)
Software	Flowjo 10.8.1
Cell population abundance	No sort was performed
Gating strategy	Cytotoxic T cells and cytokine:CD3+CD8+GZMB+; Treg cells and cytokine:CD4+CD25+FOXP3. The gating strategy was provided in the source data file.

☐ Tick this box to confirm that a figure exemplifying the gating strategy is provided in the Supplementary Information.
